# Solitary ground-glass opacity nodules of stage IA pulmonary adenocarcinoma: combination of 18F-FDG PET/CT and high-resolution computed tomography features to predict invasive adenocarcinoma

**DOI:** 10.18632/oncotarget.15577

**Published:** 2017-02-21

**Authors:** Jun Zhou, Yanli Li, Yiqiu Zhang, Guobing Liu, Hui Tan, Yan Hu, Jie Xiao, Hongcheng Shi

**Affiliations:** ^1^ Department of Nuclear Medicine, Zhongshan Hospital, Fudan University, Shanghai 200032, China; ^2^ Nuclear Medicine Institute of Fudan University, Shanghai 200032, China; ^3^ Shanghai Institute of Medical Imaging, Shanghai 200032, China

**Keywords:** positron emission tomography/computed tomography, high-resolution computed tomography, lung cancer, invasive adenocarcinoma, ground-glass opacity nodule

## Abstract

To investigate the performance of combined 18F-FDG Positron Emission Tomography/Computed Tomography with high-resolution CT for differentiating invasive adenocarcinoma from adenocarcinoma *in situ* (pre-invasive lesion) or minimally invasive adenocarcinoma in stage IA lung cancer patients with solitary ground-glass opacity nodules. This retrospective study enrolled 58 consecutive stage IA pulmonary adenocarcinoma patients with solitary ground-glass opacity nodules. The characteristics and measurements of the ground-glass opacity nodules as pure ground-glass opacity nodules and mixed ground-glass opacity nodules in the pre-invasive or minimally invasive adenocarcinoma and invasive adenocarcinoma groups on Positron Emission Tomography/Computed Tomography and high-resolution CT were compared and analyzed. Ground-glass opacity nodules in the pre-invasive or minimally invasive adenocarcinoma group preferentially manifested as pure ground-glass opacity nodule (*p* < 0.01) compared to the invasive adenocarcinoma group. While cystic appearance was more common in the invasive adenocarcinoma group (*p* < 0.05). Significant differences were found in the diameter of the ground-glass opacity nodule itself and its solid component, and consolidation/tumor ratio between the two groups. The sensitivity in predicting invasive adenocarcinoma was higher with a combined consolidation/tumor ratio > 0.38 and SUV_max_ > 1.46 in mixed ground-glass opacity nodule when compared to those of SUV_max_ > 0.95 alone or consolidation/tumor ratio> 0.39 alone (both *p* > 0.05). For a mixed ground-glass opacity nodule combined consolidation/tumor ratio > 0.38 and SUV_max_ > 1.46 appears to better predict invasive adenocarcinoma in stage IA lung cancer patients with solitary ground-glass opacity nodules.

## INTRODUCTION

Ground-glass opacity (GGO) is defined as an area of hazy increased attenuation that does not obscure underlying bronchial structures or vascular markings on high-resolution computed tomography (HRCT) [1]. Patients with stage IA lung adenocarcinoma (i.e., peripheral lung cancers ≤ 3 cm in diameter without nodal and distant metastasis), usually present as a solitary ground-glass opacity nodule (GGN) on HRCT [2–5], and have a 5-year disease-free survival rate approaching 88% [6]. On the other hand, within the stage IA lung adenocarcinoma patients, those with adenocarcinoma *in situ* or minimally invasive adenocarcinoma (AIS-MIA) have an excellent 5-year postoperative overall survival rate (100% or nearly 100%) [7, 8].

With recent advancement of technology in multi-detector row CT (MDCT), it is possible to increasingly detect small GGN and to analyze its anatomic, morphologic, and quantitative information [9–17]. Based on the presence or absence of solid components, GGN is classified into mixed GGN and pure GGN on HRCT. Although many studies have compared the morphologic appearances of different types of malignant GGNs [2, 12, 17–20], there is substantial overlap between benign GGNs and malignant GGNs [21]. Quantitative CT thus was established to evaluate GGN [9, 21, 22]. A percentage of 0.5 or less of the solid component of a GGN can identify early lung adenocarcinoma with clinical T1bN0M0 patients [13] and can be a useful independent preoperative prognostic indicator [23]. Among various strategies over morphologic evaluation, contrast-enhanced and dynamic MDCT have been applied to assess malignant GGNs with limited additional information [12]. Although perfusion MDCT shows us promising for lung cancer [24], the increased radiation dose restricts its clinical application.

Fluorin-18 fluorodeoxyglucose Positron Emission Tomography and CT (PET/CT) is increasingly used to diagnose many cancers. Higher 18F-FDG uptake can generally differentiate malignant tumors from benign lesions and normal tissue [25]. CT adds high spatial resolution value to the PET in early lung adenocarcinoma with GGN [14, 26–28]. The maximum standard uptake value (SUV_max_) of most pure GGNs and some of mixed GGNs is usually very low [29, 30]. Hence, HRCT was employed to provide additional anatomic information to PET/CT in the diagnosis of GGNs. Recently, Uehara et al. [26] reported that both SUV_max_ ≤ 2.9 and the percentage of the solid component ≤ 0.25 as well as each alone are good predictors of prognosis in patients with clinical stage IA lung adenocarcinoma. For patients with stage IA lung adenocarcinoma undergoing their first PET/CT exams, they badly need to know if they should have a limited resection or follow-up. When invasive adenocarcinoma (IAC) is suspected, no matter how many percentage of the solid component of GGNs presents, a wait-and-see CT surveillance should be terminated. The patients may achieve more benefit from a less invasive lobectomy such as robotic surgery, instead of a limited resection or other alternative treatments in patients with AIS-MIA [6, 31].

To date, the optimal performance of PET/CT with HRCT to predict IAC remains unknown. At the present study, we hypothesized that combination of consolidation/tumor ratio (CTR) and other CT features with SUV_max_ on 18F-FDG PET/CT has a good capability to differentiate IAC from AIS-MIA in malignant GGNs of stage IA lung adenocarcinoma, and it would be useful for guiding preoperative decision making in surgical resection.

## RESULTS

Readers agreement demonstrated almost perfect consistency in measurements (ICC = 0.955 for D_GGN_, ICC =0.966 for D_Solid_, and ICC = 0.996 for SUV_max_).

**Figure 1:**
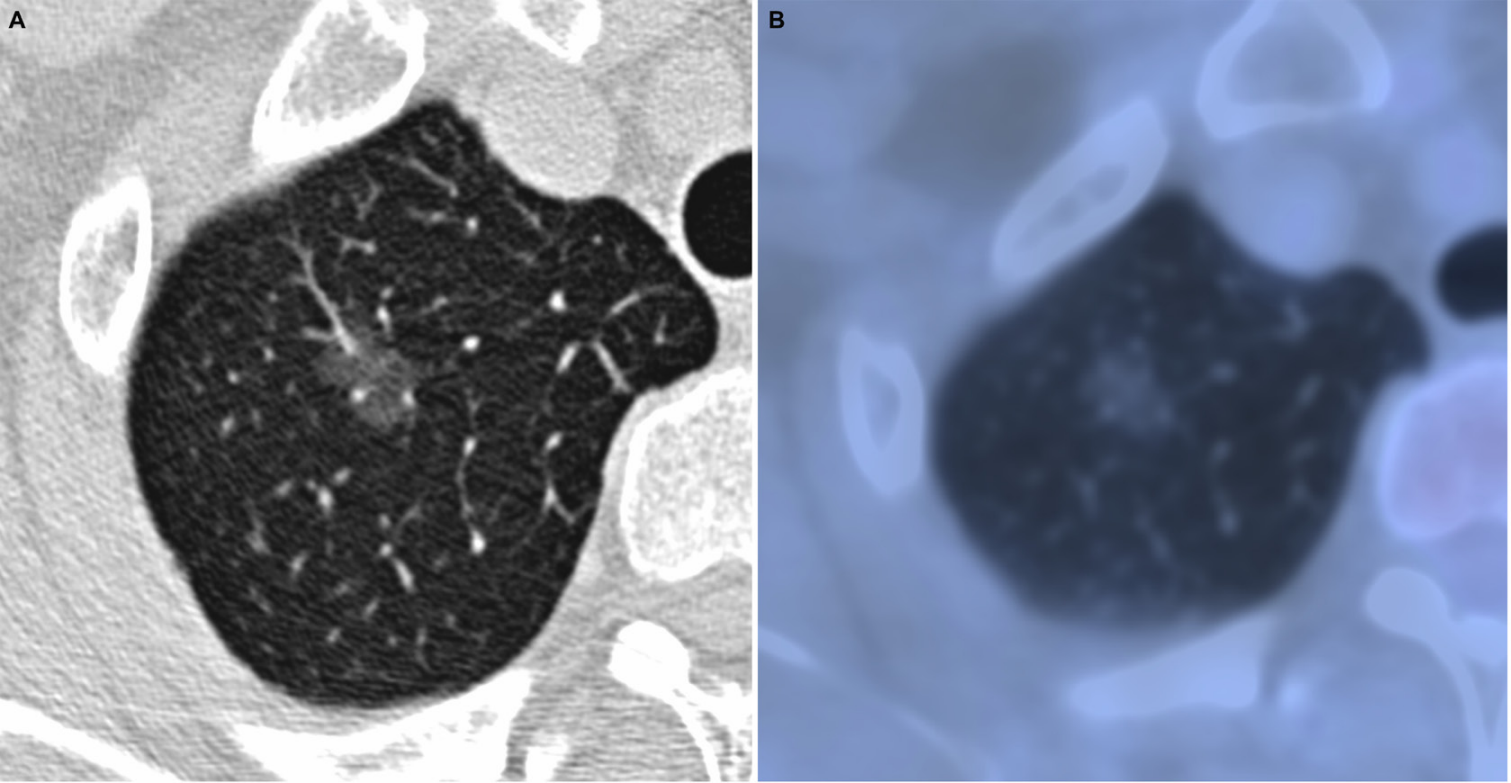
Adenocarcinoma *in situ* in 52-year-old woman (**A**) transverse lung-window HRCT scan demonstrates an oval, smooth, well-defined subpleural pure GGN in the apical segment of right upper lobe. (**B**) PET/CT fusion image shows a 12.2-mm pure GGN with no solid component and 0.67 of SUV_max_.

**Figure 2:**
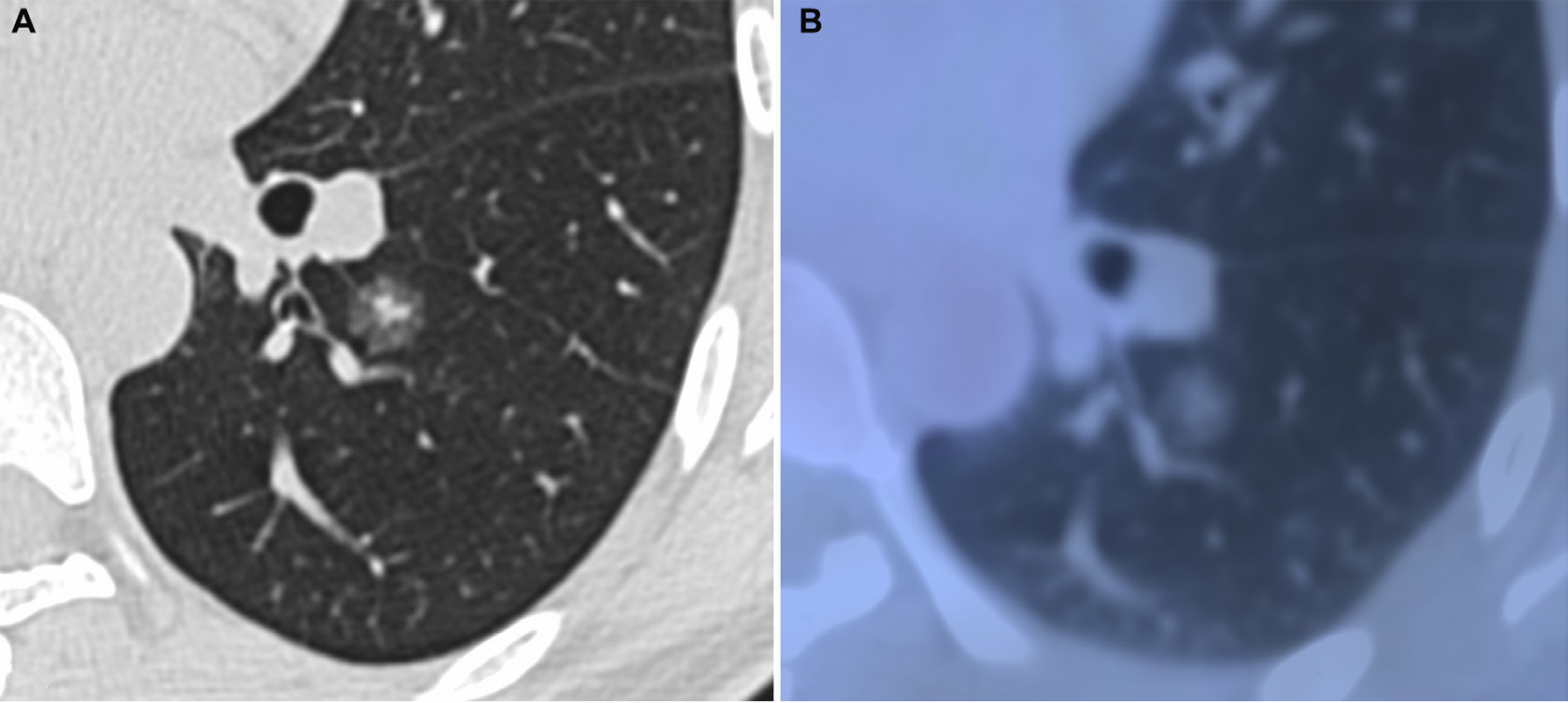
Minimally invasive adenocarcinoma in 40-year-old woman (**A**) transverse lung-window HRCT scan demonstrates a round, spiculated, well-defined parenchymal mixed GGN with pleural indentation in the apical segment of the left lower lobe. (**B**) PET/CT fusion image with perfusion mode shows a 11.9-mm mixed GGN with 0.25 of CTR and 0.81 of SUV_max_.

**Figure 3:**
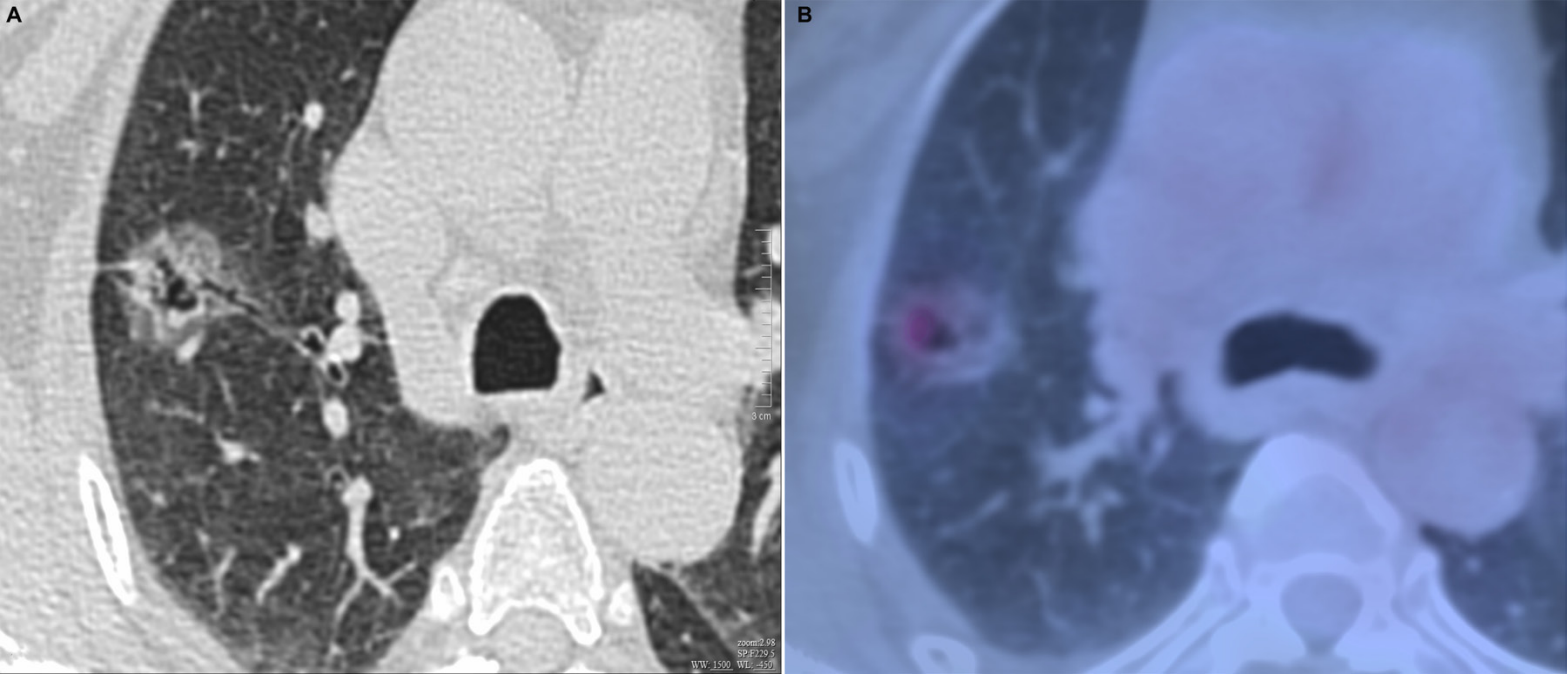
Invasive adenocarcinoma in 58-year-old woman (**A**) transverse lung-window HRCT scan demonstrates a round, lobulated, well-defined subpleural mixed GGN with natural bronchus sign, cystic appearance, and pleural indentation in the apical segment of right upper lobe. (**B**) is caudal to (A): PET/CT fusion image with perfusion mode shows a 22.5-mm oval mixed GGN with 0.68 of CTR and 3.32 of SUV_max_.

Comparisons of different pathologic diagnoses among HRCT features are displayed in Table 1. AIS-MIA cases preferentially manifested as pure GGN (*p <* 0.01) when compared to the IAC (Figure 1), while cystic appearance was more common in IAC GGNs (*p <* 0.05) (Figure 2 and Figure 3). Significant differences were also found in the D_GGN_, D_solid_, and CTR between the two groups (12.6 mm *vs* 20.0 mm, 2.3 mm *vs* 11.5 mm, 16.2% *vs* 54.5%, respectively).

**Table 1:** Morphologic features and CT measurements of solitary GGNs with stage IA lung cancer on HRCT

Features	Type	AIS-MIA (*n* = 15)	IAC (*n* = 43)	*p* Value
**Nodule Type**	**pure GGN**	8 (53.3)	2 (4.7)	0.000*
	**mixed GGN**	7 (46.7)	41 (95.3)	
**Position**	**subpleural/perifissural**	12 (80.0)	40 (83.0)	0.350*
	**parenchymal**	3 (20.0)	3 (7.0)	
**Shape**	**oval/round**	11 (73.3)	26 (60.5)	0.372*
	**irregular/polygonal**	4 (26.7)	17 (39.5)	
**Margin**	**smooth**	7 (46.7)	21 (48.8)	0.885*
	**lobulated/spiculated**	8 (53.3)	22 (51.2)	
**Bronchus sign**	**natural**	12 (80.0)	25 (58.1)	0.129*
	**dilated/distorted**	0 (0)	6 (14.0)	0.300*
	**cut-off**	0 (0)	1 (2.3)	1.000**
**Cystic appearance**	**presence**	0 (0)	12 (27.9)	0.016*
**Pleural indentation**	**presence**	6 (40.0)	38 (88.4)	0.054*
**Vascular convergence**	**presence**	12 (80.0)	37 (86.0)	1.000*
**Diameter of GGN (mm)**		12.6 ± 3.3 (8.4~20.3)	20.0 ± 5.6 (10.5~29.8)	0.000†
**Diameter of solid component (mm)**		2.3 ± 2.8 (0~8.5)	11.5 ± 6.8 (0~24.3)	0.000†
**Consolidation/tumor ratio (%)**		16.2 ± 19.3 (0~51.2)	54.5 ± 26.3 (0~94.8)	0.000‡
**CT value of GGO component (HU)**		−562.4 ± 120.0(−835.2~–327.3)	−501.9 ± 115.1(−733.0~-314.3)	0.089‡
**ΔCT_GGO-LP_ (HU)**		312.1 ± 132.5 (23.5~595.7)	370.0 ± 109.2 (171.6~575.9)	0.100‡

Note.— Data of morphologic features are expressed as number of cases with the percentages in parentheses. Data of CT measurements are expressed as mean ± standard deviation with the ranges in parentheses. GGN: ground-glass opacity nodule; HRCT: high-resolution computed tomography; AIS: adenocarcinoma *in situ*; MIA: minimally invasive adenocarcinoma; IAC: invasive adenocarcinoma; ΔCT_GGO-LP_: difference of CT value between GGO component and adjacent lung.

*Chi-square test; **Fisher`s Exact test; ^†^Mann-Whitney *U* test; ^‡^Student *t* test.

**Figure 4:**
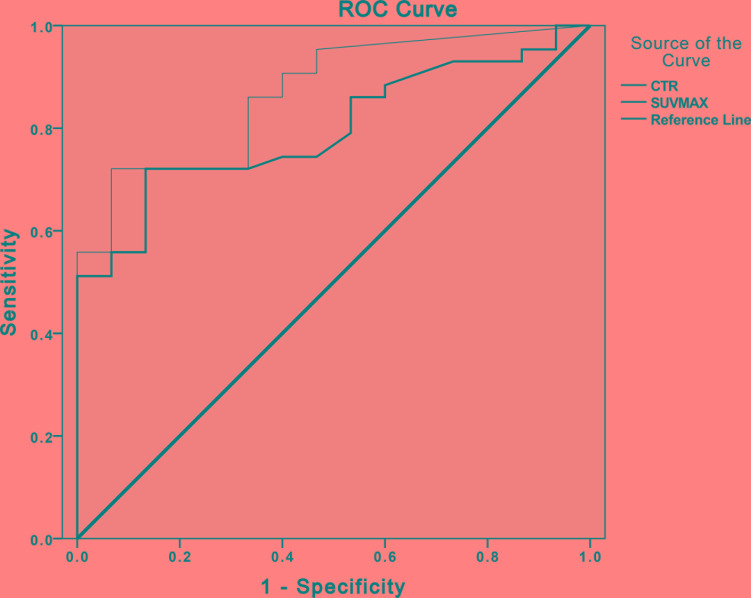
The receiver operating characteristic (ROC) curve analyses to compare the capability of SUV_max_ and CTR Graph illustrates results of ROC analyses of CTR > 0.39 (thin line) at HRCT and SUV_max_ > 0.95 (thick line) at PET/CT as reference for discriminating IAC from AIS-MIA in solitary pulmonary GGNs. Areas under ROC curve for CTR > 0.39 (0.868, *p* < 0.05) was slightly greater than that for SUV_max_ > 0.95 (0.798, *p* < 0.05). AIS: adenocarcinoma *in situ*; CTR: consolidation/tumor ratio; IAC: invasive adenocarcinoma; MIA: minimally invasive adenocarcinoma; SUV_max_: maximum standard uptake value.

The results of ROC analyses to compare the capability of SUV_max_ and CTR for differentiating IAC from AIS-MIA in solitary GGNs are displayed in Figure 4. Feasible preliminary threshold values for CTR and SUV_max_ were 0.39 and 0.95, respectively. While areas under the ROC curve were 0.868 for CTR (*p <* 0.01) and 0.798 for SUV_max_ (*p <* 0.01). According to the two cutoff values, CTR and SUV_max_ were performed respectively into binary data, which were used to control confounders of other HRCT features with statistically significant difference by binary logistic regression analysis. D_GGN_ and D_solid_ were respectively correlated with both SUV_max_ and CTR (all *p <* 0.05), whereas cystic appearance was reverse correlated with both SUV_max_ and CTR (regression coefficient = −1.609~–1.012). Hence, nodule type was the only independent HRCT characteristic parameter of no statistical significance with regression coefficient > 0, which didn't interfere with both CTR and SUV_max_.

In the present study, AIS-MIA and IAC were regarded as dependent variables, and CTR, SUV_max_, and nodule type were indicators for IAC diagnosis of solitary GGNs with stage IA lung adenocarcinoma. The corresponding multivariate logistic regression equation was as follows:

ln(*p*/1-*p*) = −3.157 + 0.984 × SUV_max_ + 0.032 × CTR + 1.108 × Nodule Type

where *p* is the probability of IAC GGN. When the *p value* was greater than or equal to 0.05, the malignant GGN was predicted to be more invasive, whereas less than 0.05 was expected to be AIS-MIA.

In terms of IAC diagnosis, the odds ratio of SUV_max_, CTR, and Nodule Type were 2.675, 1.033, and 3.027, respectively.

The exact probability values of 58 cases were calculated by backtesting the above-mentioned multivariate logistic regression equation with the specific evaluations of the SUV_max_, CTR, and Nodule Type. Afterwards, the 58 exact probability values were analyzed by ROC analyses and Youden index to determine the ultimate combined optimal cutoff values of CTR > 0.38, SUV_max_ > 1.46, and mixed GGN of Nodule Type. The capability of SUV_max_ > 0.95 alone, CTR > 0.39 alone, and the combination of CTR > 0.38, SUV_max_ > 1.46, and mixed GGN for differentiation between AIS-MIA and IAC in solitary GGNs of stage IA lung adenocarcinoma was shown in Table 2. In terms of all the performances, the ultimate combined optimal cutoff values of CTR > 0.38, SUV_max_ > 1.46, and mixed GGN demonstrated a higher sensitivity, a favourable negative predictive value and accuracy, but a balanced specificity and positive predictive value. The sensitivity of the combined CTR > 0.38, SUV_max_ > 1.46 with mixed GGN was higher than that of SUV_max_ > 0.95 alone or CTR > 0.39 alone via the McNemar test (both *p <* 0.05).

**Table 2:** Performance values of different indexes for differentiation between AIS-MIA and IAC in solitary GGNs with stage IA lung cancer on PET/CT

Index	Threshold value	Sensitivity	Specificity	PPV	NPV	Accuracy
**SUV_max_**	> 0.95	72.1 (31/43)	86.7 (13/15)	93.9 (31/33)	52.0 (13/25)	75.9 (44/58)
**CTR**	> 0.39	72.1 (31/43)	86.7 (13/15)	93.9 (31/33)	52.0 (13/25)	75.9 (44/58)
**CTR + SUV_max_ + Nodule type†**	> 0.38/> 1.46/mGGN	95.3 (41/43)*	60.0 (9/15)	87.2 (41/47)	81.8 (9/11)	86.2 (50/58)

Note.— Data are expressed as are percentages, with the numbers of solitary GGNs used to calculate the percentages in parentheses. GGN: ground-glass opacity nodule; SUV_max_: maximum standard uptake value; PPV: positive predictive value; NPV: negative predictive value; CTR: consolidation/tumor ratio.

*Significant difference (*p <* 0.05) between sensitivity of the parameter and those of SUV_max_ > 0.95 and CTR > 0.39.

^†^Combination value means that either of three parameters is positive, then the diagnosis is positive.

## DISCUSSION

According to the new adenocarcinoma classification [32], AIS and MIA are defined as small (≤ 3 cm) solitary lung adenocarcinoma with a pure or predominantly lepidic growth pattern, which means that alveolar epithelial cells are replaced by cancer cells. This growth pattern mainly manifests as a solitary GGN on HRCT and a lower FDG uptake on PET/CT [26]. Our major finding is that the combination of CTR > 0.38, SUV_max_ > 1.46, and mixed GGN was a better indicator for differentiating IAC from AIS-MIA in solitary GGNs of stage IA pulmonary adenocarcinoma.

The solid component of GGN can be central fibrosis in alveolar space, invasive component, or collapsed parenchyma [14], whereas the GGO component of GGN is formed by a lepidic growth pattern on HRCT [16]. The tendency of GGNs from non-invasive lesions, MIAs, to IACs might be corresponding to increased CTR [33]. To some extent, CTR reflected the percentage of mostly invasive solid component within GGN on HRCT. In terms of GGN with very low SUV_max_ [29, 30], the widely used threshold value of SUV_max_ > 2.5 to predict malignant tumor in clinical practice is not powerful any more. This is because that higher SUV_max_ values may predict more highly invasive tumors on PET/CT [33]. In this investigation, we combined CTR with SUV_max_ and HRCT features to determine an optimal threshold to differentiate IAC from AIS-MIA. Our result revealed that the combination of CTR > 0.38, SUV_max_ > 1.46, and mixed GGN yielded a higher sensitivity (95.3%) and accuracy (86.2%) than the SUV_max_ > 0.95 (72.1% and 75.9%) to predict IAC. Our result was consistent with the previously published report [14, 33, 34]. None of the patients with pre-invasive AIS showed that both SUV_max_ ≤ 1.0 and CTR ≤ 0.40 had lymphatic vessel invasion [14]. According to some CT-based reports, CTR > 0.53 is helpful for predicting IAC among peripheral lung adenocarcinomas within 3 cm in diameter [33], while CTR > 0.62 might predict invasiveness of stage IA lung cancer from AIS-MIA [34]. Furthermore, the previous studies showed that peripheral or clinical T1b lung adenocarcinomas with CTR ≤ 0.5 were nonaggressive adenocarcinomas with better outcomes [4, 13]. Therefore, our study could provide more precise clue preoperatively to stop a further CT surveillance, employ a standard or less invasive lobectomy, or even prepare an alternative treatment for patients with incomplete tumor resection by predicting IACs. On the other hand, those patients predicting as non-IAC would furtherly differentiate MIA from AIS, and they may plan carefully to have a limited resection or a follow-up CT scan.

CT features of GGNs may reflect their potential malignant nature. In our study, binary logistic regression analyses showed that only nodule type did not interfere with both CTR and SUV_max_. Our data were consistent with previous reports [16, 17], that mixed GGN was more common within IAC GGN. Recently, Kakinuma et al. [35] found that at least a small portion (approximately 1%) of solitary pure GGNs within 5 mm will develop into IACs or MIAs appearing as solitary mixed GGNs. Thereby, increased SUV_max_ and CTR with mixed GGN may predict solitary GGNs developing into more invasive. There was significant difference of D_GGN_ and D_solid_ between IAC and AIS-MIA, which is consistent with previous study [17]. There was also markedly statistical significance of cystic appearance between IAC and AIS-MIA, it may be the reason that lung cancer has an association with cystic appearance [36] and especially there was significant difference in cystic appearance between pure GGN and mixed GGN [37]. However, these excluded HRCT features interfered with both SUV_max_ and CTR via binary logistic regression analyses. Furthermore, there was no statistic difference of position, shape, margin, bronchus sign, pleural indentation, vascular convergence, CT_GGO_, and ΔCT_GGO-LP_ between IAC and AIS-MIA groups. These findings are consistent with the previous studies [16, 17, 38].

There are several limitations in our present study. First, selection bias may occur because some stage IA lung adenocarcinoma patients with a solitary GGN were excluded due to the lack of PET/CT examination before operation. Second, this study has a small sample size, a further prospective investigation with a large number of cases is needed to verify the present predicative mode.

## MATERIALS AND METHODS

### Patients

This study was approved by the institutional review board of our hospital. We retrospectively reviewed all stage IA lung adenocarcinoma patients in our hospital between June 2010 and May 2016. All patients must meet the following inclusion criteria: (i) adenocarcinoma or its precursor; (ii) a solitary pulmonary nodule (SPN) with ground glass opacity; (iii) 3 cm or smaller of the lesion size; (iv) preoperative PET/CT; (v) preoperative focal continuous HRCT examination; (vi) surgical resection; and (vii) within one month interval of all PET/CT, HRCT, and GGN resection. This investigation excluded these kind of subjects: (i) the diameter of a solitary GGN ≥ 3 cm; (ii) multiple nodules; (iii) poor image quality due to motion artifact; (iv) with any anti-tumoral therapies; (v) adenocarcinoma exceeding stage IA; and (vi) direct evidence of synchronous primary or prior malignancy in the past 5 years.

Consecutive fifty-eight patients with a mean age of 60.2 years ± 9.5 (range 39~81 years; M: F = 18: 40) were included our study. The interval between final PET/CT with HRCT and surgery ranged from 1 to 25 days (4.4 days ± 4.0). Of the 58 GGNs, mixed GGN was seen in 48 cases, and pure GGN in 10 cases.

According to the new IASLC/ATS/ERS adenocarcinoma classification [31], pathologic diagnoses included AIS in 3 cases, MIA in 12 cases, and IAC in the rest 43 cases (acinar predominant in 32 cases, lepidic predominant in 6 cases, papillary predominant in 4 cases, and invasive mucinous in one case). The cases were divided into two groups based on clinical implication of treatment: AIS-MIA group and IAC group.

### 18F-FDG PET/CT examination and image analysis

All patients fasted for at least 6 hours prior to PET/CT study. Insulin was discontinued at least 6 hours before examination, and a serum blood glucose level was verified to be below 10 mmol/L. All patients received FDG intravenously from 333 to 481 MBq, and then rested quietly around 60 minutes.

The following two dedicated diagnostic PET/CT devices were employed: Discovery VCT unit (GE Medical Systems, Waukesha, USA) and uMI S-96R unit (United Imaging, Shanghai, China) with a 64/16-MDCT scanner. Low-dose registration CT and a whole-body PET were acquired from head to mid-thigh. All PET/CT scans were displayed via a uWS-MI R001 workstation (United Imaging, Shanghai, China) and lung (window width, 1000 HU; window level, -700 HU) and mediastinal (window width, 300 HU; window level: 45 HU) window settings.

Two nuclear medicine physicians (1 year and 10 years of experience) who were blind to all of the clinical data and pathologic diagnosis, evaluated the PET/CT images by joint review. For a semi-quantitative analysis of FDG uptake, a large oval volume of interest (VOI) was used. The SUV_max_ of a GGN was measured in an original size or a fit size with standard lung window and PET perfusion mode, and the GGN was fully encased. All measurements were performed with 2 times zoom. When misregistration was found between PET and CT images, manual correct of the PET/CT was made. All measurements were performed 3 times and averaged.

### High-resolution CT scanning and image analysis

All patients underwent a breath-hold HRCT scan immediately after routine PET/CT scanning at our hospital. The scope of continuous HRCT scans was obtained only for pulmonary nodules region with the patients in a supine position. Two aforementioned MDCT scanners were used with the following settings: GE/UI scanners [120 kVp; 500 mA/200 mAs; tube rotation time, 0.4~1 s per rotation; pitch, 0.937~1.375; slice thickness, 0.5~0.625 mm; reconstruction algorithm, Bone/B_VSHARP_C; and matrix, 512×512]. All HRCT scans were displayed via a uWS-MI R001 workstation and lung (window width, 1500 HU; window level, -450 HU) and mediastinal (window width, 300 HU; window level: 45 HU) window settings.

Two radiologists (with 6 years and 12 years of experience in chest CT image analysis) who were unaware of all the clinical data and pathologic diagnosis, assessed the CT feature of GGN in position (subpleural/perifissural and parenchymal), type (pure GGN and mixed GGN), shape (round/oval and polygonal/irregular), margin (smooth and lobulated/spiculated), internal characteristics (bronchus sign and cystic appearance), and adjacent structures (pleural indentation and vascular convergence). Bronchus sign was classified into natural, dilated and distorted, and cut-off [39]. Cystic appearance was defined as an oval, round, or large area of low attenuation within GGNs [40]. In cases of discrepancies between the two radiologists, a consensus was reached by joint review.

The diameter of GGN (D_GGN_) was as the longest diameter on the transverse standard lung window image. For a mixed GGN, the diameter of the solid component (D_Solid_) was also measured in the longest diameter as the D_GGN_ on the transverse standard lung window image. The attenuation value of the GGO component (CT_GGO_) and normal lung parenchyma adjacent to GGO (CT_LP_) were measured using an oval region of interest covering the identical objects. Vessels, bronchi, and air-containing space within GGO were spared as far as possible, and lung markings were away from measurement of normal lung parenchyma adjacent to GGO.

All of the measurements were carried out independently by two radiologists using electronic calliper with 3~8 times zoom, whereas vascular convergence was evaluated with original or fit size. Then, the measurements were averaged. The ratio of the maximum diameter of solid component to the maximum tumor diameter (consolidation/tumor ratio, CTR) was calculated as previously reported [14]. According to HRCT feature, CTR of a pure GGN = 0, whereas CTR of a mixed GGN < 1.0 but > 0. The difference of CT_GGO_ and CT_LP_ (ΔCT_GGO–LP_) was calculated as follows: CT_GGO –_CT_LP_.

### Statistical analysis

All numerical values are reported as mean ± standard deviation (mean ± SD). Statistical analyses were performed by using software (IBM SPSS version 22.0 for Mac OS, IBM, USA). All variables with a value of *p <* 0.05 were considered to indicate statistically significant differences.

Intraclass correlation coefficient (ICC) [41] was used to estimate consistency of measurements made by two nuclear medicine physicians and radiologists.

To determine the differences of HRCT features of the GGNs between AIS-MIA and IAC, we performed the Chi-square test or Fisher`s exact test for qualitative data and the student *t* test or Mann-Whitney *U* test for quantitative data.

We also used ROC analysis and Youden index to identify the feasible preliminary threshold values of SUV_max_ at PET/CT and CTR at HRCT for distinguishing invasive adenocarcinoma from pre-invasive or minimally invasive adenocarcinoma in stage IA patients with solitary GGNs. Subsequently, binary logistic regression analysis was employed to exclude potential confounders of other HRCT features with statistical significant difference or regression coefficient < 0 on the basis of binary SUV_max_ and CTR data, respectively.

Then, a logistic regression equation including SUV_max_, CTR, and other HRCT features was formed by multivariate logistic regression analysis. The three included parameters with their specific evaluations or measurements of the 58 patients were backtested by the newly formed logistic regression equation to compute 58 exact probability values, which were used to determine an optimal probability value by ROC analysis and Youden index. According to the optimal probability value, the ultimate combined optimal cutoff values of the SUV_max_, CTR, and other HRCT features were identified to predict invasion of stage IA lung adenocarcinoma with GGO component. The positive diagnosis (i.e., invasive adenocarcinoma) was established by either of any positive combined optimal cutoff values. Finally, the sensitivity, specificity, positive predictive value, negative predictive value, and accuracy among the preliminary threshold values of SUV_max_ or CTR, and the ultimate combined optimal cutoff values of the SUV_max_, CTR, and other HRCT features were compared via the McNemar test to differentiate IAC from AIS-MIA in stage IA lung adenocarcinoma patients with solitary GGNs.

## CONCLUSIONS

In conclusion, a mixed GGN with the combination of CTR > 0.38 and SUV_max_ > 1.46 may be more reliably to predict IAC from AIS-MIA in stage IA lung adenocarcinoma patients with solitary GGNs.
