# Correction: Solitary ground-glass opacity nodules of stage IA pulmonary adenocarcinoma: combination of 18F-FDG PET/CT and high-resolution computed tomography features to predict invasive adenocarcinoma

**DOI:** 10.18632/oncotarget.26671

**Published:** 2019-02-05

**Authors:** Jun Zhou, Yanli Li, Yiqiu Zhang, Guobing Liu, Hui Tan, Yan Hu, Jie Xiao, Hongcheng Shi

**Affiliations:** ^1^ Department of Nuclear Medicine, Zhongshan Hospital, Fudan University, Shanghai 200032, China; ^2^ Nuclear Medicine Institute of Fudan University, Shanghai 200032, China; ^3^ Shanghai Institute of Medical Imaging, Shanghai 200032, China

**This article has been corrected:** The ‘p’ value in the second-last sentence of the Abstract section has been changed. The corrected text of the Abstract is shown below. The authors declare that these corrections do not change the results or conclusions of this paper.

**ABSTRACT**

**To investigate the performance of combined 18F-FDG Positron Emission Tomography/Computed Tomography with high-resolution CT for differentiating invasive adenocarcinoma from adenocarcinoma *in situ* (pre-invasive lesion) or minimally invasive adenocarcinoma in stage IA lung cancer patients with solitary ground-glass opacity nodules. This retrospective study enrolled 58 consecutive stage IA pulmonary adenocarcinoma patients with solitary ground-glass opacity nodules. The characteristics and measurements of the ground-glass opacity nodules as pure ground-glass opacity nodules and mixed ground-glass opacity nodules in the pre-invasive or minimally invasive adenocarcinoma and invasive adenocarcinoma groups on Positron Emission Tomography/Computed Tomography and high-resolution CT were compared and analyzed. Ground-glass opacity nodules in the pre-invasive or minimally invasive adenocarcinoma group preferentially manifested as pure ground-glass opacity nodule (*p* < 0.01) compared to the invasive adenocarcinoma group. While cystic appearance was more common in the invasive adenocarcinoma group (*p* < 0.05). Significant differences were found in the diameter of the ground-glass opacity nodule itself and its solid component, and consolidation/tumor ratio between the two groups. The sensitivity in predicting invasive adenocarcinoma was higher with a combined consolidation/tumor ratio > 0.38 and SUV**_**max**_ **> 1.46 in mixed ground-glass opacity nodule when compared to those of SUV**_**max**_ **> 0.95 alone or consolidation/tumor ratio> 0.39 alone (both *p* < 0.05). For a mixed ground-glass opacity nodule combined consolidation/tumor ratio > 0.38 and SUV**_**max**_ **> 1.46 appears to better predict invasive adenocarcinoma in stage IA lung cancer patients with solitary ground-glass opacity nodules.**

Original article: Oncotarget. 2017; 8:23312-23321. https://doi.org/10.18632/oncotarget.15577

